# Impact of Coronavirus Disease 2019 on Inpatient Palliative Care Services in Japan: A Hybrid Time-Series Analysis Before, During, and After the Pandemic Using 10-Year Claims Data

**DOI:** 10.1177/26892820251385778

**Published:** 2025-10-07

**Authors:** Yuki Egashira, Syo Nakamura, Naoki Suzuki, Ryo Watanabe

**Affiliations:** ^1^ Graduate School of Health Innovation, Kanagawa University of Human Services, Kawasaki-shi, Kanagawa, Japan.; ^2^ Cancer Prevention and Control Division, Kanagawa Cancer Center Research Institute, Yokohama-shi, Kanagawa, Japan.; ^3^ Department of Clinical Oncology, Yamagata Faculty of Medicine, Yamagata University, Yamagata-shi, Yamagata, Japan.

**Keywords:** claims data, COVID-19, inpatient palliative care, Japan, time-series analysis

## Abstract

**Background:**

Coronavirus disease 2019 (COVID-19) reduced the use of routine medical care, particularly the treatment of nonlife-threatening conditions. Previous studies have examined the impact of the COVID-19 pandemic; however, most focused on the effect of early stages, lacking data on the later stages and post-pandemic recovery.

**Objective:**

To examine the impact of COVID-19 on inpatient palliative care services in Japan throughout the pandemic and after its reclassification as a Category 5 infectious disease.

**Design:**

Retrospective time-series analysis of claims data from April 2014 to March 2024.

**Setting/Subjects:**

National Health Insurance participants in Kanagawa Prefecture who received the “Palliative Care Unit Inpatient Fee” or “additional fee for inpatient palliative care treatment.”

**Measurements:**

The observed-to-expected (OE) ratios were calculated using Seasonal Autoregressive Integrated Moving Average model predictions. Interrupted time-series (ITS) analysis was used to examine the level and trend changes after the reclassification of COVID-19 to Category 5 (May 2023).

**Results:**

During COVID-19, the Palliative Care Unit Inpatient Fee showed OE ratios ranging from –25.1% to 22.9% (April 2020–November 2021), which eventually reached –36.0% (October 2022). After COVID-19 reclassification, OE ratios improved to –9.5% to 12.5%. Additional fees for inpatient palliative care treatment showed similar patterns, with the lowest point being –42.7% (February 2022). The ITS analysis revealed significant positive trends for both services after reclassification.

**Conclusions:**

The COVID-19 pandemic has influenced inpatient palliative care services, particularly since its sixth wave. The reclassification of COVID-19 to Category 5 showed immediate positive effects, suggesting that policy interventions can effectively restore palliative care services. These findings highlight the vulnerability of palliative care, impact of policy interventions during pandemics, and need for strategic planning in a future-aging society.

## Key Message

COVID-19 has significantly reduced the number of inpatient palliative care services in Japan, particularly since the sixth wave. However, the reclassification of COVID-19 to Category 5 in May 2023 showed immediate positive effects, demonstrating that policy interventions can effectively restore palliative care services during pandemic recovery.

## Background

In March 2020, the World Health Organization (WHO) declared coronavirus disease 2019 (COVID-19) a pandemic. Various movement restrictions were implemented in 186 countries, ranging from interregional travel advisories to mandatory lockdowns in 82 countries.^1,2^ Simultaneously, the postponement of nonurgent treatment and prioritization of COVID-19 treatment were recommended.^3^ Previous studies have reported a reduction in elective surgeries during the pandemic, with approximately 28 million surgeries postponed or canceled worldwide during the 12-week peak period.^4^ This indicates that COVID-19 significantly affects routine medical care, especially the treatment of nonlife-threatening conditions.

Few studies have reported the impact of COVID-19 on palliative care. A Taiwanese study found that hospice ward occupancy rates decreased from 81.6% to 71.8% during the early pandemic stages.^5^ A study of Indian tertiary hospitals reported a sharp decline in palliative care during the 2020 lockdown compared with the previous year.^6^ Although COVID-19 may have affected palliative care provision, its established role in improving quality of life and family satisfaction remains critical, especially with estimated global needs for serious health-related conditions expected to double by 2060 compared with 2016, mainly in low- and middle-income countries.^7–9^

Palliative care is a part of Japan’s universal health insurance system. This system restricts palliative care services to patients with cancer, Human Immunodeficiency Virus, or end-stage heart failure, which differs from many Western countries where palliative care encompasses a broader range of life-limiting conditions, including neurological, cardiac, and respiratory diseases, as well as COVID-19. To control the spread of COVID-19, the government issued a statement in April 2020 to prevent cross-prefecture movement and postpone deferrable surgeries. Previous studies have reported decreased elective surgeries in the early stages of the pandemic.^10,11^ Similarly, surveys by the Japanese Society for Hospice and Palliative Care found that palliative care services were also affected, with 15% and 20% of hospitals in the first and second surveys, respectively, reporting partial or complete closure of palliative care wards to accommodate patients with COVID-19.^12,13^ Conversely, no reductions in palliative care team interventions were reported in general wards.^14^ Thus, the impact of the COVID-19 pandemic on palliative care in Japan varied according to the reporting entity and survey. Clarifying this impact in Japan, with its aging population, will reveal useful implications for future infectious disease outbreaks in Japan and other aging countries.

Previous palliative care studies conducted during the COVID-19 pandemic have limitations. Many studies have only investigated the early stages of the pandemic and have lacked data on the impact of the Delta strain (summer 2021), Omicron strain (early 2022), and subsequent outbreaks. The impact of COVID-19 after the WHO lifted the health care emergency in May 2023 also remains unknown. In addition, comparisons were made only for the same month of the previous year, and the long-term trend impacts were unknown. Finally, these studies were limited to specific facilities and had limited generalizability.

To overcome these limitations, this study examined the long-term impact of COVID-19 on inpatient palliative care services in Japan throughout the pandemic period, including the Delta and Omicron variant phases, and evaluated the effect of policy intervention (reclassification as a Category 5 infectious disease in May 2023). We performed a time-series analysis using large-scale real-world data (public insurance claims data) spanning 10 years, including pre-pandemic data and post-Category 5 reclassification data, to examine the impact of COVID-19 beyond the early stages, including Delta and Omicron strain phases, and the effect of reclassification of COVID-19 to Category 5 under the Infectious Disease Control Law.

We hypothesized that (1) inpatient palliative care services would experience sustained reductions during later pandemic waves, particularly during the Delta and Omicron variant periods, similar to patterns observed in the initial outbreak; and (2) the reclassification of COVID-19 to Category 5, which removed strict health care delivery restrictions, would result in the immediate recovery of palliative care service provision to pre-pandemic levels.

## Methods

### Study design and study population

#### Study design

This retrospective study used the claims data of patients with inpatient palliative care service calculations from April 2014 to March 2024. We defined and analyzed three periods: pre-COVID-19 (April 2014–March 2020), COVID-19 (April 2020–April 2023), and post-COVID-19 (May 2023–March 2024).

#### Population

We chose Kanagawa Prefecture for several reasons: it has Japan’s second-largest population after Tokyo, making it highly representative;^15^ it was the first region in Japan with positive COVID-19 cases, suggesting a potentially greater impact on inpatient palliative care than in other regions;^16^ and it has Japan’s lowest per capita hospital beds,^17^ hypothetically increasing the impact on inpatient palliative care.

The study participants were individuals with National Health Insurance in Kanagawa Prefecture from April 2014 to March 2023, with confirmed “Palliative Care Unit Inpatient Fees” or “additional fee for inpatient palliative care treatment.” Table 1 presents the definitions of these fees.

**Table 1.** Service Types in This Study

Service fee name	Ward type	Service definition
Palliative care unit inpatient fee (A310)	Palliative care unit	Fees for services with admission to a palliative care unit that meets facility standards for malignant tumors and acquired immunodeficiency syndrome
Additional fee for inpatient palliative care treatment (A226-2)	General unit	Fees for services with palliative care teams in general hospital beds. It is calculated when a patient with a malignant tumor, an immunodeficiency syndrome, or end-stage heart failure is admitted to a general hospital bed, while presenting with physical symptoms such as pain, fatigue, or dyspnea, or psychiatric symptoms, (such as anxiety or depression), is treated by a team for symptom relief based on patient consent

In Japan, all citizens have medical insurance coverage, with the National Health Insurance covering approximately 18.0% of those aged 0–64 years and 68.5% of those aged 65–74 years.^18^

### Data sources

We used medical fee data from the Kokuho Database System (KDB), which contains medical fee billing data for insured Japanese individuals with national insurance, including single proprietors and retired individuals. The medical claims data contained de-identified patient information, including visit month, treatment number, insurance number, hospital number, and diagnosis. The KDB has been used in various research fields, including epidemiology, with confirmed data reliability.^19,20^

### Outcomes

First, we examined the difference between predicted and actual palliative care service values after April 2020 using the observed-to-expected (OE) ratio, which was evaluated by plotting the peak months of COVID-19 cases in the resulting figure.^21^ Wave definitions were based on Kanagawa Prefecture reports,^22^ focusing primarily on the peak infection months in each wave (1^st^, 2020/4; 3^rd^, 2021/1; 5^th^, 2021/8; 6^th^, 2022/2; 7^th^, 2022/8; and 8^th^, 2022/12). Peaks for the 2^nd^ and 4^th^ waves were not defined because of flat infection trends. For palliative care ward admission fees, we calculated the aggregate bed values reported by medical institutions.^23^

Second, the level and trend changes in interrupted time-series (ITS) were set as outcomes. In both cases, National Health Insurance participant numbers were converted to services per million insured people based on Kanagawa Prefecture public data.^24^ As the number of insured individuals was publicly available only through FY2022, we used the FY2014–FY2022 average percentage change for FY2023 values (April 2023–March 2024). Municipalities with missing data were excluded.

### Statistical analyses

First, a Seasonal Autoregressive Integrated Moving Average (SARIMA) analysis was performed to estimate the data for April 2020 to March 2024 based on actual data from April 2014 to March 2020.

SARIMA comprises an autoregressive component using historical data for forecasting, a difference (I) component removing data trends, moving average component using historical forecast errors, and seasonal component indicating seasonal patterns. The SARIMA model is (*p*,d,q) × (*P*,*D*,*Q*,*m*), where *p*, *d*, and *q* are the ARIMA model parameters; *P*, *D*, *Q*, and *m* are seasonal component parameters; and *m* is the seasonality period.

We estimated the trends in the number of patients receiving inpatient palliative care using data for 72 months (April 2014–March 2020).

Model selection followed the following procedures: (1) The Dickey–Fuller test assessed stationarity, with significance set at *p* < 0.05. (2) The arimaauto package selected the lowest Akaike information criterion among the candidate (*p*, *d*, *q*) × (*P*, *D*, *Q*, *m*) parameters.^25^ (3) A white noise test using the Ljung–Box chi-squared test was performed on the selected models, with the significance set at *p* > 0.05. Below this value, the SARIMA model parameters were reset until the white noise exceeded 0.05. (4) The mean absolute percentage error (MAPE) between the predicted and measured values from April 2014 to March 2020 was calculated to evaluate the fit of the final model.

Second, the ITS analysis analyzed trends and slopes during the COVID-19 (April 2020–April 2023) and post-COVID-19 Category 5 transition (May 2023–March 2024) regarding infectious disease reclassification and institutional reforms. Category 5 is a Japan-specific infectious disease classification requiring reporting but not isolation measures. The ITS analysis measured two time-series data changes: (1) level change (outcome value change immediately post-intervention) and (2) trend change (outcome change rate difference before and after the intervention). This evaluated both “immediate” and “sustained” intervention effects. This method has been used in previous studies on the impact of emergency declarations on elective medical treatment.^11,26^ Statistical significance was set at *p* < 0.05.

StataMP 16.1 was used for statistical analyses.

### Ethical considerations

The Research Ethics Review Committee of the Graduate School of Health Innovation of the Kanagawa University of Human Services approved this study (Approval No.: SHI No. 71).

## Results

We identified eight municipalities with missing months; therefore, 48 of 56 municipalities were included in our analysis. Table 2 lists the basic characteristics of palliative care services before COVID-19 (April 2014–March 2020), during the pandemic (April 2020–April 2023), and after the pandemic (May 2023–March 2024).

**Table 2.** Basic Characteristics of Palliative Care Services during Different Periods

	Before COVID-19 (2014/4–2020/3)	During COVID-19 (2020/4–2023/4)	Post COVID-19 (2023/5–2024/3)	*p-*value
Palliative Care Unit Inpatient Fee (A310)				
*N*	9330	4209	1326	
Age^*1^	66.7	67.3	67.2	<0.01
SD	7.2	7.1	7.3	
Sex (%)^*2^				<0.01
Male	53.1%	51.8%	56.8%	
Female	46.9%	48.3%	43.2%	
Admission days per month^*1^	14.3	13.6	14.0	<0.01
SD	9.9	9.8	10.1	
Additional fee for inpatient palliative care treatment (A226-2)				
* N*	7291	4070	1464	
Age^*1^	64.5	65.3	65.2	0.03
SD	8.9	8.7	9.2	
Sex (%)^*2^				0.12
Male	51.8%	53.6%	53.7%	
Female	48.2%	46.4%	46.3%	
Admission days per month^*1^	9.6	7.8	7.7	<0.01
SD	7.7	6.6	6.6	

^*1^ One-way analysis of variance.

^*2^ Chi-squared test.

COVID-19, coronavirus disease; SD, standard deviation.

Comparing these periods, the average age significantly differed for the Palliative Care Unit Inpatient Fee (A310) and additional fee for inpatient palliative care treatment (A226-2). Male patients predominated both services; however, this was only significant for palliative care hospitalization. The number of admission days per month significantly differed among periods for both the Palliative Care Unit Inpatient Fee (A310) and additional fee for inpatient palliative care treatment (A226-2).

**FIG. 1.**
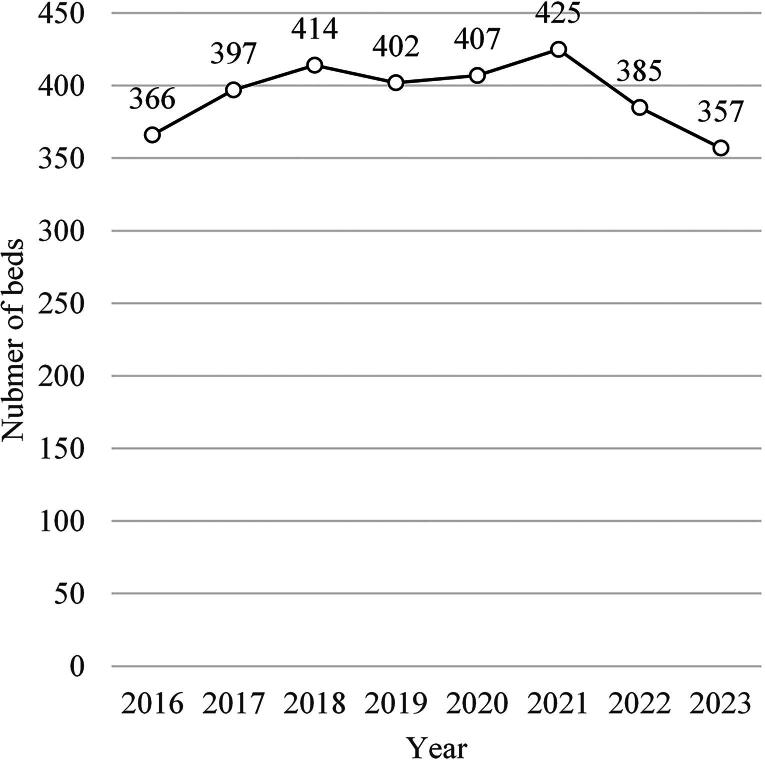
Number of inpatient bed numbers in palliative care wards.

Figure 1 shows the number of inpatient beds in the palliative care wards of Kanagawa Prefecture hospitals, revealing an increasing trend from 2016 to 2021 and a decreasing trend after 2022.

**FIG. 2.**
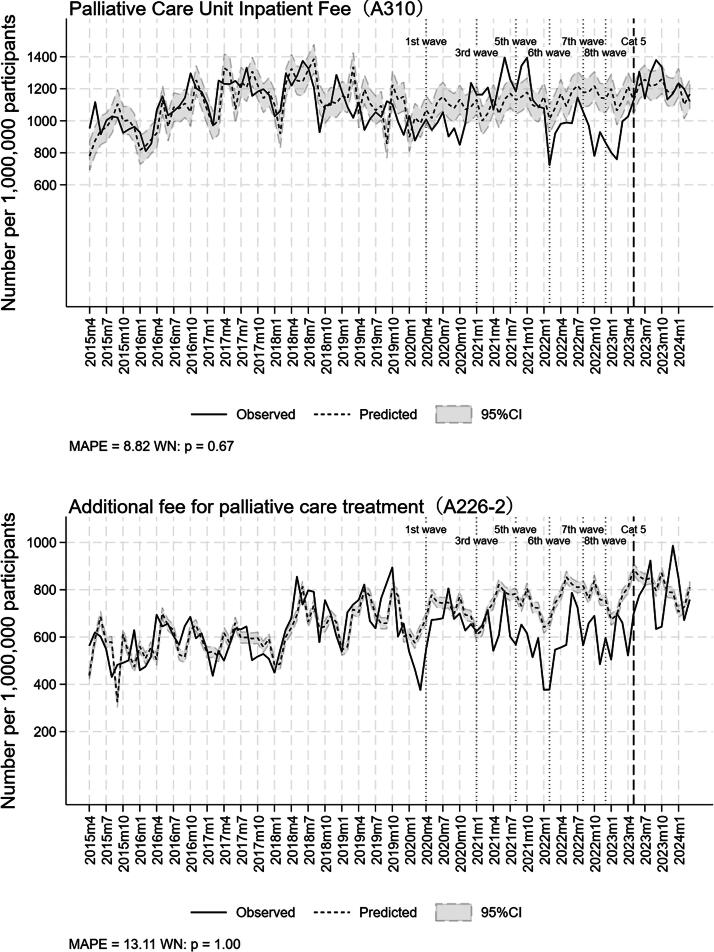
Results of Seasonal Autoregressive Integrated Moving Average (SARIMA) model analysis.

Figure 2 shows the measured and predicted values over time for the palliative care ward Palliative Care Unit Inpatient Fee (A310) and additional fee for inpatient palliative care treatment (A226-2).

**FIG. 3.**
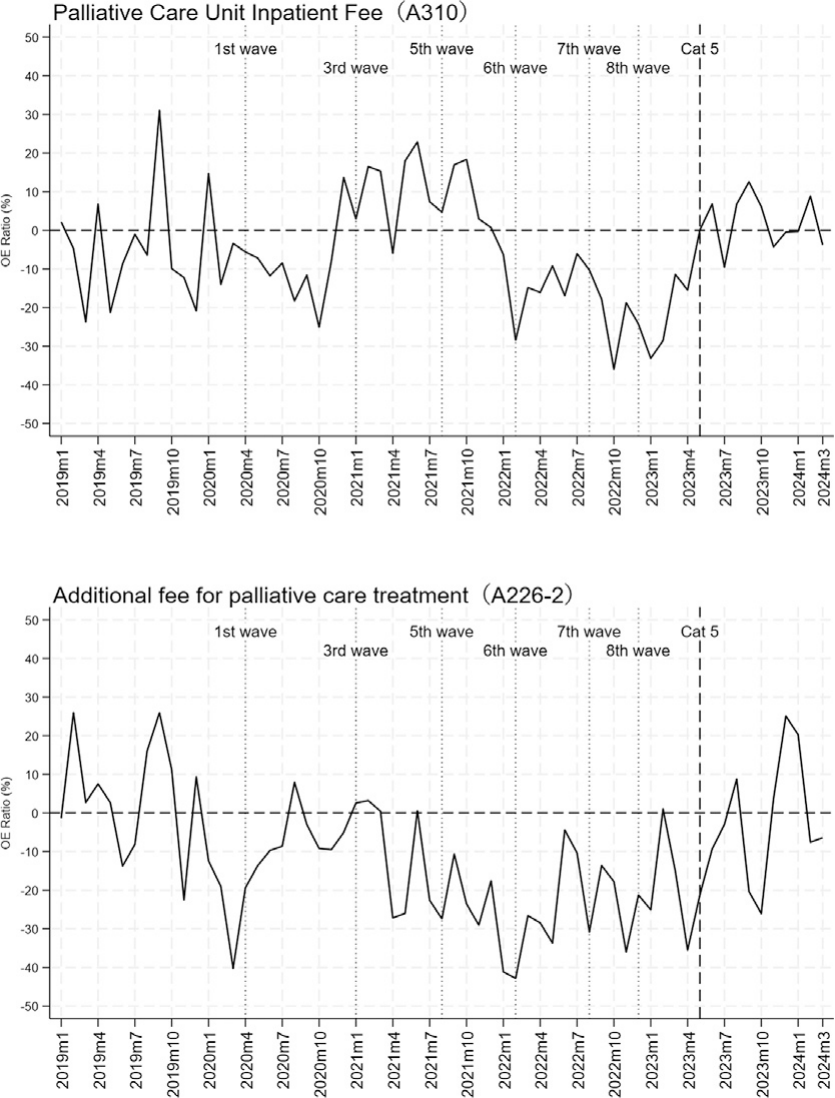
Results of observed-to-expected ratio calculations.

The dotted lines represent the fitted values obtained using the SARIMA model. Before April 2020, these were in-sample fitted values, and after April 2020, we extended the same fitted values as out-of-sample projections owing to technical limitations, because StataMP produced errors when attempting to calculate proper forecast confidence intervals with our parameter specifications. In typical SARIMA forecasting, confidence intervals widened progressively over the forecast horizon to reflect increasing uncertainty. However, this limitation does not affect the point estimates or interpretation of trends. Figure 3 demonstrates the trend in the OE ratio. The MAPEs of the prediction model were 8.82% for palliative care ward admission fees and 13.11% for additional palliative care treatment fees.

Regarding the OE ratio, the Palliative Care Unit Inpatient Fee (A310) fluctuated between –25.1% and 22.9% from April 2020 to November 2021. Furthermore, from November 2021 to April 2023 (pre-Category 5 transition), it showed a consistent negative trend, reaching the lowest value of –36.0% in October 2022. In addition, it was –28.5% during the sixth peak wave (February 2022). After the May 2023 Category 5 transition, the OE ratio increased from –9.5% to 12.5% through March 2024.

The OE ratio of the additional fee for inpatient palliative care treatment (A226-2) fluctuated between –19.4% and 7.9% from April 2020 to March 2021 and from July 2021 to April 2023 (pre-Category 5 transition), showing a primarily negative trend, reaching the lowest point of –42.7% in February 2022 (sixth wave peak). After the May 2023 Category 5 transition, the values showed a wide range, from –26.1% to 25.12% through March 2024.

**FIG. 4.**
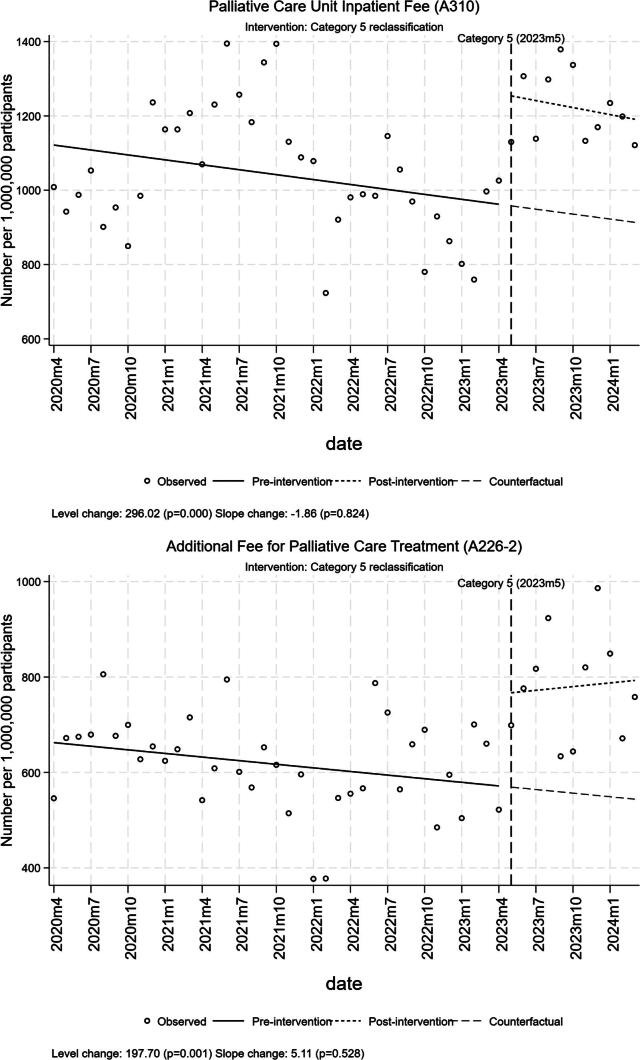
Results of interrupted time-series analysis with intervention of reclassification of COVID-19 to Category 5 disease.

Figure 4 displays the ITS analysis results for the impact of Category 5 transition on the Palliative Care Unit Inpatient Fee (A310) and additional fee for inpatient palliative care treatment (A226-2).

The results indicated a positive and significant trend for both services. The additional fee for inpatient palliative care treatment (A226-2) had a positive value, although the change in slope was not significant.

## Discussion

This study clarified the impact of the COVID-19 pandemic and post-Category 5 transition effects using two time-series analyses: the SARIMA model and ITS analysis.

### Findings

First, at the beginning of the COVID-19 pandemic, both the Palliative Care Unit Inpatient Fee (A310) and additional fee for inpatient palliative care treatment (A226-2) temporarily fell below predicted values following the first emergency declaration in April 2020. However, the actual results equaled or exceeded the predicted values until the fifth wave peak in August 2021. From the sixth wave onward, actual values remained consistently below predicted levels.

Several factors may have contributed to this post-sixth wave of decline. First, as shown in Figure 1, the number of palliative care beds gradually decreased from 425 in 2020 to 385 in 2022 and 357 in 2023. This trend is consistent with the findings of a Japan Hospice Palliative Care Association survey: among 51 facilities that closed or partially closed palliative care wards for patients with COVID-19 by November 2021, 21 (75%) planned to remain closed after December 2021.^13^ Regarding infection numbers in Kanagawa Prefecture, the peak number of infected individuals (7-days moving average) during the August 2021 Delta strain outbreak exceeded 2500, whereas during February 2022, new infections exceeded 7200 daily, nearly three times more.^27^ Consequently, increased COVID-19 admissions to general hospital beds likely caused a reduction in admissions of patients with cancer. The following reasons may explain this: other patients were eligible for additional palliative care treatment, reduction in transfers from general wards to palliative care wards, palliative care team reallocation to the COVID-19 response, and conversion of palliative care wards into COVID-19 beds.

Patient-related factors may also explain the persistent gap between actual and predicted palliative care utilization. A study conducted at 37 Japanese facilities in August 2021 (fifth wave) found that among 33 patients, many chose home care due to pandemic-related visitation restrictions at inpatient facilities.^28^ In addition, in a study of 72 bereaved families, 52.8% reported that the COVID-19 pandemic influenced their care decisions, with many citing visitation restrictions. Therefore, patients and families may have chosen home care over inpatient palliative care due to pandemic-related hospital visitation limitations.^29^ A combination of hospital- and patient-related factors likely caused a consistent downward trend after the sixth wave.

Although these factors are unclear, we can infer that the COVID-19 pandemic has demonstrated vulnerability to inpatient palliative care provision. Palliative care needs are likely to increase in the near future, considering the aging population in Japan and globally. This implies that future emerging infectious disease outbreaks may generate larger palliative care hospitalization waiting lists that coincide with pandemic peaks. To address these challenges, measures are required to accommodate patients requiring care while working within the constraints of hospital beds.

Second, the ITS analysis results showed an immediate upward effect of the Category 5 infectious disease transition on both the Palliative Care Unit Inpatient Fee (A310) and additional fee for inpatient palliative care treatment (A226-2). This suggests that easing medical care provision and nonurgent care restrictions induced an immediate intervention effect. For the Palliative Care Unit Inpatient Fee, post-May 2023 transition OE ratios generally remained within ±10%, similar to that of the pre-COVID-19 pandemic trends. Therefore, the end of the policy intervention influenced the downward trend in palliative hospitalization. Although an immediate upward effect was observed for the additional fee for inpatient palliative care treatment (A226-2) with palliative care team intervention, the subsequent OE ratio ranged within 30%, which differed from the pre-COVID-19 pandemic-estimated trends. This may reflect the impact of palliative care team reassignment, intervention system changes, or differing needs between patients in palliative care wards and those requiring additional palliative care treatment.

Ethical considerations are required before postponing nonlife-saving medical treatment during emerging infectious disease outbreaks such as COVID-19. Palliative care involves ethical considerations that differ from quantitative life-saving. It aims to improve patients’ quality of life and death, enabling meaningful living during their limited time remaining, although it does not save quantitative life. These ethical dilemmas require thorough discussion in the future planning of infectious disease outbreak management.

### Limitations

This study has some limitations. First, the generalizability of its findings is limited because it was only conducted in a single prefecture (Kanagawa). In addition, the claims data of the residents of Kanagawa Prefecture did not include out-of-prefecture patients such as those who lived in the Tokyo metropolitan area and visited Kanagawa medical institutions. Future generalizability improvements require multi-prefecture comparisons and national data analyses using resources such as national databases.

Second, there may be population bias because we included participants from the National Health Insurance system. This system includes nonworkers and retired individuals who potentially represent a lower-income population. A lower sociodemographic status is associated with barriers to palliative care utilization.^30^ Therefore, our findings may have been underestimated.

Third, although the rapid decrease in care provision, especially during the sixth wave, correlated with the rapidly increasing number of infections, a clear causal relationship remains unclear. Future clarification requires interviews with palliative care-related medical institutions, patients, and families.

Finally, although this study identified a declining trend in inpatient palliative care during infectious disease outbreaks, the findings did not necessarily indicate palliative care recession. In Japan, home palliative care services are provided, but data limitations have prevented a long-term trend analysis of such services. Therefore, understanding the overall transformation of the palliative medical system requires an analysis of the impact of home-based palliative care.

Despite these limitations, this study used 10 years of long-term claims data to comprehensively identify service volume changes in inpatient palliative care during and after the pandemic, based on pre-COVID-19 data. It also showed an immediate recovery to pre-pandemic levels through the policy intervention of transitioning COVID-19 to a Category 5 infectious disease, particularly for palliative care ward admissions.

## Conclusion

This study investigated the impact of the COVID-19 pandemic and its resolution on inpatient palliative care using 10-year long-term data. The findings revealed declining inpatient palliative care provision during the surge of emerging COVID-19 and an immediate upward trend in provision following the policy intervention of transitioning COVID-19 to Category 5 infectious disease.

## Abbreviations Used

COVID-19: Coronavirus Disease 2019
WHO: World Health Organization
KDB: Kokuho Database System
OE ratio: Observed-to-Expected ratio
ITS: Interrupted Time-Series
SARIMA: Seasonal Autoregressive Integrated Moving Average
ARIMA: Autoregressive Integrated Moving Average
MAPE: Mean Absolute Percentage Error
FY: Fiscal Year
SD: Standard Deviation

## References

[1] Han E, Tan MMJ, Turk E, et al. Lessons learnt from easing COVID-19 restrictions: An analysis of countries and regions in Asia Pacific and Europe. Lancet 2020;396(10261):1525–1534. 32979936 10.1016/S0140-6736(20)32007-9 PMC7515628

[2] Don’t let children be the hidden victims of COVID-19 pandemic. 2020. Available from: https://www.unicef.org/press-releases/dont-let-children-be-hidden-victims-covid-19-pandemic [Last accessed: June 4, 2025].

[3] COVIDSurg Collaborative. Global guidance for surgical care during the COVID-19 pandemic. Br J Surg 2020;107(9):1097–1103. 32293715 10.1002/bjs.11646 PMC7262310

[4] COVIDSurg Collaborative. Elective surgery cancellations due to the COVID‐19 pandemic: Global predictive modelling to inform surgical recovery plans. Br J Surg 2020;107(11):1440–1449; doi: 10.1002/bjs.11746 32395848 PMC7272903

[5] Chou YC, Yen YF, Feng RC, et al. Impact of the COVID-19 pandemic on the utilization of hospice care services: A cohort study in Taiwan. J Pain Symptom Manage 2020;60(3):e1–e6; doi: 10.1001/jama.2016.16840 PMC7831889 32663615

[6] Karthik A, Rustagi K, Mishra S, et al. Effect of nation-wide lockdown on palliative care services in a Tertiary Care Centre in India: A retrospective observational study. Indian J Palliat Care 2020;26 (Suppl 1):S45–S47; doi: 10.4103/ijpc.ijpc_142_20 33088086 PMC7535002

[7] Kavalieratos D, Corbelli J, Zhang D, et al. Association between palliative care and patient and caregiver outcomes: A systematic review and meta-analysis. JAMA 2016;316(20):2104–2114; doi: 10.1001/jama.299.14.1698 27893131 PMC5226373

[8] Zimmermann C, Riechelmann R, Krzyzanowska M, et al. Effectiveness of specialized palliative care: A systematic review. JAMA 2008;299(14):1698–1709. 18398082 10.1001/jama.299.14.1698

[9] Sleeman KE, Brito M D, Etkind S, et al. The escalating global burden of serious health-related suffering: Projections to 2060 by world regions, age groups, and health conditions. Lancet Glob Health 2019;7(7):e883–e892; doi: 10.1016/S2214-109X(19)30172-X 31129125 PMC6560023

[10] Japanese Society of Surgery. Recommendations for surgical procedures for patients with positive or suspected new coronavirus (revised). 2020. Available from: https://jp.jssoc.or.jp/modules/aboutus/index.php?content_id=53 [Last accessed: June 4, 2025].

[11] Okuno T, Takada D, Shin J h, et al. Surgical volume reduction and the announcement of triage during the 1st wave of the COVID-19 pandemic in Japan: A cohort study using an interrupted time series analysis. Surg Today 2021;51(11):1843–1850; doi: 10.1007/s00595-021-02286-6 33881619 PMC8059122

[12] The Japanese Society for Hospice and Palliative Care. Results of the 1st Questionnaire Survey on the impact of COVID-19 in palliative care units. 2021. Available from: https://www.hpcj.org/info/covid19/covid19_pcuchosa202103.pdf [Last accessed: June 4, 2025].

[13] The Japanese Society for Hospice and Palliative Care. Results of the Second Questionnaire Survey on the impact of COVID-19 in palliative care units. 2021. Available from: https://www.hpcj.org/info/covid19/covid19_pcuchosa202111.pdf [Last accessed: June 4, 2025].

[14] Kurachi A, Hamada H, Tanoue T, et al. Five years of activity in our Palliative Care Team and Coronavirus disease. Palliat Care Res 2023;18(1):73–77; doi: 10.2512/jspm.18.73

[15] Statistics Bureau Ministry of Internal Affairs and Communications. Population estimates/current population estimates as of October 1, 2023. Available from: https://www.stat.go.jp/english/data/jinsui/2023np/index.html [Last accessed: June 4, 2025].

[16] Amengual O, Atsumi T. COVID-19 pandemic in Japan. Rheumatol Int 2021;41(1):1–5; doi: 10.1007/s00296-020-04744-9 33205224 PMC7671668

[17] Ministry of Health, Labour and Welfare. Overview of the 2022 medical facilities (dynamic) survey and hospital report (in Japanese). 2022. Available from: https://www.mhlw.go.jp/toukei/saikin/hw/iryosd/22/ [Last accessed: June 4, 2025].

[18] Ministry of Health, Labour and Welfare. National Health Insurance Survey FY2022 summary of survey results (in Japanese). 2022. Available from: https://www.e-stat.go.jp/stat-search/files?page=1&layout=datalist&toukei=00450397&tstat=000001214140&cycle=8&tclass1=000001214141&result_page=1&tclass2val=0 [Last accessed: June 4, 2025].

[19] Narita N, Okumura K, Kinjo T, et al. Trends in prevalence of non-valvular atrial fibrillation and anticoagulation therapy in a Japanese region ― analysis using the national health insurance database. Circ J 2020;84(5):706–713; doi: 10.1253/circj.CJ-18-0989 32213724

[20] Nagai K, Iseki C, Iseki K, et al. Higher medical costs for CKD patients with a rapid decline in eGFR: A cohort study from the Japanese general population. PLoS One 2019;14(5):e0216432–e11; doi: 10.1371/journal.pone.0216432 31100069 PMC6524806

[21] Ministry of Health, Labour and Welfare. Survey on the state of medical care and the number of inpatient beds (in Japanese). Available from: https://www.mhlw.go.jp/stf/seisakunitsuite/newpage_00023.html [Last accessed: June 4, 2025].

[22] Kanagawa Prefectural Government. New coronavirus infection Kanagawa prefecture response record (in Japanese). 2023. Available from: https://www.pref.kanagawa.jp/docs/ga4/covid19/archive/records.html [Last accessed: June 4, 2025].

[23] Ministry of Health, Labour and Welfare. Reporting of hospital bed functions. Available from: https://www.mhlw.go.jp/stf/seisakunitsuite/bunya/0000055891.html [Last accessed: June 4, 2025].

[24] Kanagawa Prefectural Government. National Health Insurance business status (in Japanese). Available from: https://www.pref.kanagawa.jp/docs/n5p/cnt/f7093/p1128867.html [Last accessed: June 4, 2025].

[25] Bolotov I. ARIMAAUTO: Stata module to find the best ARIMA model with the help of a Stata-adjusted Hyndman-Khandakar (2008) algorithm. 2022. Available from: https://ideas.repec.org/c/boc/bocode/s459043.html [Last accessed: June 4, 2025].

[26] Shah SA, Brophy S, Kennedy J, et al. Impact of first UK COVID-19 lockdown on hospital admissions: Interrupted time series study of 32 million people. EClinicalMedicine 2022;49:101462; doi: 10.1016/j.eclinm.2022.101462 35611160 PMC9121886

[27] Ministry of Health, Labour and Welfare. National data base (open data). Available from: https://www.mhlw.go.jp/stf/covid-19/open-data.html [Last accessed: June 4, 2025].

[28] Hamano J, Tachikawa H, Takahashi S, et al. Changes in home visit utilization during the COVID-19 pandemic: A multicenter cross-sectional web-based survey. BMC Res Notes 2022;15(1):238; doi: 10.1186/s13104-022-06128-7 35799212 PMC9261221

[29] Iida T, Ito N, Okamura N, et al. Home care in the era of COVID-19—Results from the bereaved families of terminal cancer patients survey. Palliat Care Res 2023;18(1):55–60; doi: 10.2512/jspm.18.55

[30] Lewis JM, DiGiacomo M, Currow DC, et al. Dying in the margins: Understanding palliative care and socioeconomic deprivation in the developed world. J Pain Symptom Manage 2011;42(1):105–118; doi: 10.1016/j.jpainsymman.2010.10.265 21402460

